# Sarcopenia Prediction for Elderly People Using Machine Learning: A Case Study on Physical Activity

**DOI:** 10.3390/healthcare11091334

**Published:** 2023-05-05

**Authors:** Minje Seok, Wooseong Kim

**Affiliations:** Computer Engineering Department, Gachon University, Seongnam 13120, Gyeonggi, Republic of Korea

**Keywords:** sarcopenia, physical activity, machine learning, elderly, KNHANES

## Abstract

Sarcopenia is a well-known age-related disease that can lead to musculoskeletal disorders and chronic metabolic syndromes, such as sarcopenic obesity. Numerous studies have researched the relationship between sarcopenia and various risk factors, leading to the development of predictive models based on these factors. In this study, we explored the impact of physical activity (PA) in daily life and obesity on sarcopenia prediction. PA is easier to measure using personal devices, such as smartphones and watches, or lifelogs, than using other factors that require medical equipment and examination. To demonstrate the feasibility of sarcopenia prediction using PA, we trained various machine learning models, including gradient boosting machine (GBM), xgboost (XGB), lightgbm (LGB), catboost (CAT), logistic regression, support vector classifier, k-nearest neighbors, random forest (RF), multi-layer perceptron, and deep neural network (DNN), using data samples from the Korea National Health and Nutrition Examination Survey. Among the models, the DNN achieved the most precise accuracy on average, 81%, with PA features across all data combinations, and the accuracy increased up to 90% with the addition of obesity information, such as total fat mass and fat percentage. Considering the difficulty of measuring the obesity feature, when adding waist circumference to the PA features, the DNN recorded the highest accuracy of 84%. This model accuracy could be improved by using separate training sets according to gender. As a result of measurement with various metrics for accurate evaluation of models, GBM, XGB, LGB, CAT, RF, and DNN demonstrated significant predictive performance using only PA features including waist circumference, with AUC values at least around 0.85 and often approaching or exceeding 0.9. We also found the key features for a highly performing model such as the quantified PA value and metabolic equivalent score in addition to a simple obesity measure such as body mass index (BMI) and waist circumference using SHAP analysis.

## 1. Introduction

The significant loss of skeletal muscle mass is one of the representative senescence-related diseases in the elderly. Currently, 50 million people worldwide have sarcopenia, accounting for approximately 13% of individuals aged 60–70 years [1], and the global population over 60 years is expected to double by 2050 [2,3]. Sarcopenia not only degrades the life quality of the elderly but induces socioeconomic problems. Furthermore, chronic musculoskeletal disorders from sarcopenia cause substantial health problems such as mortality, dyslipidemia, physical disability, metabolic disorder, falls, and fractures.

South Korea is one of the fast-aging countries, as the elderly population is anticipated to increase drastically from 5.3 M (15.7% of the whole population) in 2022 to 19 M (40% of the whole population) in 2050 [4,5]. Accordingly, sarcopenia will threaten the public health system of South Korea significantly in the near future. The Ministry for Health, Welfare & Family Affairs recently assigned a disease code to sarcopenia in Korean Standard Classification of Diseases and now encourages related research.

According to the survey of Korea National Health and Nutrition Examination Survey (KNHANES) conducted from 2008 to 2011, the prevalence of class 2 sarcopenia among older adults in South Korea, defined by muscle mass according to height, was 12.4% for men and 0.1% for women. Many studies on the relation between sarcopenia and various factors in the Korean elderly population have been conducted using the KNHANES. In [6,7], the relation between sarcopenia and cardiovascular disease was explored. The association of sarcopenia with the pulmonary function was studied in [8,9,10,11], and sarcopenic obesity and metabolic syndrome were also studied in [12,13,14]. Both high levels of PA and a high ratio of energy intake to basal metabolic rate were independently associated with a lower risk of sarcopenia, according to [15]. Some life activities, such as long sleep duration [16] and walking [17], are associated with sarcopenia.

As highlighted in the aforementioned studies, sarcopenia is a multifactorial disease with poor nutrition, inflammatory pathway activation, neurological decline, low-level PA, hormonal changes, fatty infiltration, and chronic illness [18]. In this study, we focused on PA as a key risk factor for sarcopenia, which is defined as age-related muscle fiber loss that typically starts around the age of 50 and results in the loss of approximately 50% of muscle fibers by the age of 80 years [19,20]. In addition, obesity is correlated with PA, which causes discomfort and severely interferes with PA, eventually leading to a lack of PA [21,22]. As more PA can stave off the development of sarcopenia and obesity in the elderly [23], sarcopenia prediction based on abundant data on PA and obesity can help the elderly recognize their risk of sarcopenia.

Machine learning (ML) is useful for establishing a prediction model for sarcopenia. Kang et al. [24] applied ML techniques such as logistic regression (LR), random forest (RF), gradient boosting machine (GBM), and support vector classifier (SVC) with features of nutritional factors to sarcopenia prediction. According to the results, the prediction accuracy of the models was comparable, with an area under the receiver operating characteristic (ROC) curve (AUC) value of 0.8. Meanwhile, key features for the prediction were body mass index (BMI) and parathyroid hormone rather than nutrition intake. Kim et al. [25] used ophthalmological examinations and demographic factors for ML prediction of sarcopenia. The algorithms used were LR, XGBoost, and TabNet, a type of deep learning, and an AUC value of approximately 0.74 was obtained.

We developed a sarcopenia prediction model using PA-related information that is easy to obtain compared with other information measured by special devices and experts. To construct the model, we used several ML techniques, including LR, SVC, K-nearest neighbors (KNN), ensemble learning bagging-type RF, and boosting-types such as GBM, XGBoost (XGB), LightGBM (LGB), and CatBoost (CAT). In addition, we considered a deep learning model using MLP and deep neural networks (DNNs).

We obtained an accuracy on average of 90% for the model trained with PA and obesity features across all data combinations but approximately 81% without obesity features. Considering the difficulty of measuring obesity features, we used only waist circumference information, which is easy to measure, as one of the obesity features. Therefore, the accuracy increased to 84%, and it was further improved when using separate training sets for each gender. Its AUC as a reliable performance evaluation measure was over 0.85 for the GBM, XGB, LGB, CAT, RF, and DNN models. Although there were differences by gender, the AUCs often approached or exceed 0.9 in these models.

To know how much an input variable affects a model, eXplainable Artificial Intelligence (XAI) has received significant attention, enabling the explanation of the operation and final result of the deep learning model. SHAP [26] is one of the XAI based on the Shapley value [27,28], originating from cooperative game theory, which can numerically express how much each feature contributes to the model output. In addition, by scoring the features with high importance across models, features are evaluated in terms of impact on model learning. As a consequence, we observed that the PA features such as metabolic equivalent (MET) score, moderate PA, and walking, in addition to general information, such as age, BMI, and waist circumference, play a key role in all the aforementioned models.

The remainder of this paper is organized as follows. We briefly explain the muscle mass measurement methodology and sarcopenia diagnosis with cutoff values and describe the dataset for this study in Section 2. In Section 3, we introduce PA and obesity features used for ML and show the statistical analysis of these features for sarcopenia. We introduce several ML techniques for a comparison study in Section 4 and present the experimental results and reliability of model in Section 5. Further discussion about feature selection is presented in Section 6. Finally, we conclude in Section 7.

## 2. Materials and Methods

### 2.1. Muscle Mass Measurement Techniques

For diagnosing sarcopenia, the measurable indicators include muscle mass, muscle strength, and physical performance; specifically, we have to measure the amount of muscle and its function. In this study, we focus on muscle mass among these to diagnose sarcopenia.

The European Working Group on Sarcopenia in Older People (EWGSOP) has provided guidelines on the technique and suitability of measuring parameters for the diagnosis of sarcopenia. EWGSOP suggests body imaging techniques and bioelectrical impedance analysis (BIA) [29] to assess muscle mass. Various imaging techniques are available for estimating muscle mass or lean body mass, including computed tomography (CT), magnetic resonance imaging (MRI), and dual-energy X-ray absorptiometry (DXA). CT and MRI can obtain extremely precise muscle mass values, but there are concerns about the high cost and radiation exposure. Therefore, as an alternative with minimal radiation exposure, DXA is the preferred measurement method for research. BIA is an inexpensive test for estimating fat mass and lean mass and can be applied not only to ambulatory patients but also to bedridden patients. Furthermore, BIA results have a good correlation with MRI predictions [30]. Accordingly, BIA can be a good portable alternative to DXA.

### 2.2. Sarcopenia Diagnosis and Cutoff Values

This section introduces the muscle mass measurement methods used in previous studies. The criteria for diagnosing sarcopenia with muscle mass using the aforementioned measurement technique and its cutoff value have also been presented in several studies.

Baumgartner et al. [31] first defined the diagnostic criteria using DXA, where the appendicular skeletal muscle mass (ASM) was measured by excluding the mass of bones in the extremities and then by further excluding the fat mass. The increase in limb muscle mass against height is corrected by dividing by the square of height such as BMI. Such an ASM/ht${}^{2}$(kg/m${}^{2}$) value is used as the diagnostic criteria for sarcopenia. The cutoff values were established by determining values that were 2σ (standard deviation) below the mean values for young adults aged 18–40 years in the Rosetta Study reference data: 7.26 for men and 5.45 for women.

Janssen et al. [30,32] used a formula that considers the values measured by BIA, height squared, gender, and age to estimate muscle mass: skeletal muscle mass (kg) = [(height${}^{2}$/BIA-resistance × 0.401) + (gender × 3.825) + (age ×−0.071)] + 5.102. This research demonstrated a robust association between MRI-based measurement of skeletal muscle mass and the resistance index (Ht${}^{2}$/R) obtained through BIA, indicating that BIA is a reliable technique for estimating skeletal muscle mass. Limited evidence suggests that there is a strong correlation between appendicular BIA measurements and ASM, as assessed by single CT and DXA. They transformed the absolute measurement of skeletal muscle mass (kg) to percentage skeletal muscle mass (muscle mass/body mass × 100) called the skeletal muscle index (SMI; ASM (kg)/body weight (kg) × 100), which considers a stature and the mass of nonskeletal muscle tissues such as fat, organ, and bone. To consider body composition components other than height and muscle, the percentage value for weight between −1σ and −2σ of the young reference group was considered class I sarcopenia. A case of subjects whose SMI was below −2σ was defined as class II sarcopenia.

Newman et al. [33] assessed participants using DXA and used two methods to diagnose sarcopenia. The first index was calculated as the ratio of appendicular lean mass (aLM) to height squared, and the second index was a measure of aLM that was adjusted for both height and body fat mass (using residuals). In the first method, rather than using a cutoff from a younger population, which Baumgartner et al. had classified in the past, to compare this method with an alternative, participants were categorized as having sarcopenia if their index value was within the lowest 20% of the distribution for their gender. The cutoff points used in this study, 7.23 kg/ht${}^{2}$ for men and 5.67 kg/ht${}^{2}$ for women, were comparable to those reported in previous studies, which were 7.26 kg/ht${}^{2}$ for men and 5.45 kg/ht${}^{2}$ for women. In the second method, they employed linear regression analysis to investigate the association between aLM with both height (m) and fat mass (kg). Sarcopenia was defined on the basis of the cutoff value of the 20th percentile of the distribution of residuals. The previous approach showed a strong correlation with BMI and tended to classify fewer overweight or obese individuals as sarcopenic. Using the aLM/ht${}^{2}$ method, no sarcopenic cases were detected among obese individuals in both men and women, whereas the residual method identified 11.5% of men and 21.0% of women in the obese group as sarcopenic.

An operational definition and diagnostic strategy [29] for sarcopenia proposed by EWGSOP has been the most commonly used worldwide. However, the cutoff value based on the European population is unsuitable for Asians because of variations in lifestyles, cultural backgrounds, body sizes, and ethnicities. Therefore, we adopted an Asian consensus on the diagnosis of sarcopenia [34] that the Asian Working Group for Sarcopenia (AWGS) developed. We considered the index of muscle mass among muscle mass, muscle strength, and physical performance for cutoff values in this study. The AWGS suggests determining the cutoff value for muscle mass using −2σ of the mean muscle mass for a young reference group, or using the lower quintile of the population. They present indicators for DXA and BIA figures, which are cost-effective and simple compared with other methods. They recommend using DXA to measure height-adjusted skeletal muscle mass instead of weight-adjusted skeletal muscle mass, with cutoff values of 7.0 kg/m${}^{2}$ in men and 5.4 kg/m${}^{2}$ in women for diagnosing low muscle mass. Furthermore, the cutoff values measured by BIA for diagnosing low muscle mass are 7.0 kg/m${}^{2}$ in men and 5.7 kg/m${}^{2}$ in women, based on the ASM/height${}^{2}$.

### 2.3. Participants and Data Selection

This study was based on the KNHANES gathered by the Korea Centers for Disease Control and Prevention under the Korea National Health and Nutrition Examination Ministry of Health and Welfare and used a stratified, clustered, multistage probability sampling design to monitor the public health of the Korean population from 2008 to 2011. We considered surveys carried out only from 2008 to 2011 containing measurements of bone density and body fat for the sarcopenia diagnosis as a most recent data set. The KNHANES involves three components: a health interview, a health examination, and a dietary survey. The health examination involved body measurements, blood pressure and pulse measurement, chest X-ray examination, bone mineral density and body fat examination, etc. The body measurement was performed by well-trained examiners using automatic body measurement equipment and DXA after removal of all personal items such as shoes, jewelry, and waist belt, but not paper gowns, from participants. The health interview included information about smoking, alcohol consumption, PA, mental health, comorbidity, educational and economic status, and health-related quality of life. There were more than 500 variables for a health questionnaire and laboratory findings; the survey collected data through standardized physical examinations performed in specially equipped mobile examination centers and include household interviews conducted with the participants.

**Figure 1:**
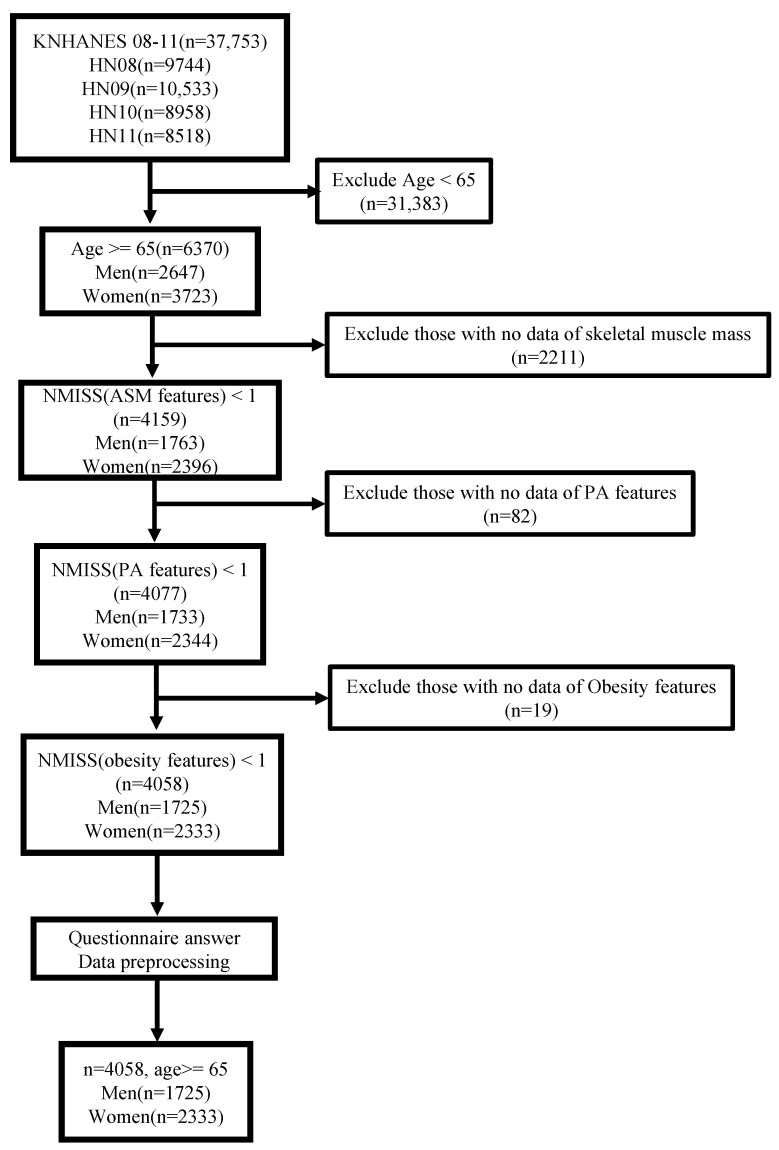
Process of data selection, Korea National Health and Nutrition Examination Survey (KNHANES) IV and V (2008–2011).

First, we selected samples on the bone mineral density and body fat tests required to predict sarcopenia in the survey, in which the bone mass content and bone mass density were assessed by the difference in DXA radiation transmittance using low-energy penetrating soft tissue and high-energy radiation penetrating bone tissue. A total of 37,753 participants from the KNHANES were included in the data samples in this study. For the age of diagnosis of sarcopenia based on the definition of the elderly in each country, as mentioned by AWGS [34], we first considered the population over the age of 65 year, which is specified for the elderly in the Welfare of the Elderly Act of Korea. Accordingly, participants under the age of 65 years (n = 31,383) were excluded, and additional participants without skeletal muscle mass information for sarcopenia diagnosis were excluded as well (n = 2211), as depicted in Figure 1. To establish a sarcopenia prediction model using ML based on PA and obesity features, we excluded participants without PA features (n = 82), such as number of days of walking per week, and subsequently obesity features (n = 19) such as BMI. As a result, we extracted 4058 participants from the original population.

## 3. Feature Analysis

In this section, we introduce features related to PA and obesity used for our prediction model and explain their implication and derivation methods. Furthermore, we analyze the feature data statistically with respect to sarcopenic tendencies.

### 3.1. Features of Physical Activity

PA was evaluated with the Korean short version of the International Physical Activity Questionnaire (IPAQ) [35] on PA over the last seven days, which demonstrates excellent measurement characteristics. The IPAQ was developed for cross-country PA and physical inactivity monitoring measurement, and multinational reliability and validity have been demonstrated through many tests.

In detail, the surveyed features in the KNHANES include the number of days of vigorous PA per week (BE3_11), the duration (minutes) of vigorous PA (BE3_12), the number of days of moderate PA per week (BE3_21), the duration (minutes) of moderate PA (BE3_22), the number of walking days per week (BE3_31), and the walking duration (minutes) (BE3_32). The vigorous PA rate (pa_high) refers to practicing for at least 20 min per day for more than 3 days a week, and moderate PA rate (pa_mid) and walking rate (pa_walk) indicate doing each activity for a minimum of 30 min per day for more than 5 days a week. The number of days of flexibility exercise per week (BE5_2) and the number of days of strength exercise per week (BE5_1) are also included.

For additional information, the provided PA features are processed to generate IPAQ features. Comparable estimates of PA can be quantified by a continuous score (MET-minutes/week) by calculating MET values according to each activity type and classified into High, Moderate, and Low according to the above features. Average MET values are derived from the compendium for each activity [36]. According to the IPAQ data, the MET values for walking, moderate PA, and vigorous PA are 3.3, 4.0, and 8.0, respectively. The continuous score can be calculated by adding all of each activity type-MET value × the number of days performed during the week × the number of minutes usually performed per day.

To be classified as a High activity level, individuals must meet one of these two criteria: (i) participate in vigorous-intensity activity for at least 3 days per week with a minimum score of 1500 MET-minutes/week or (ii) participate in any combination of walking and moderate- or vigorous-intensity activity for 7 or more days per week, with a minimum score of 3000 MET-minutes/week. To be classified as a Moderate activity level, individuals must meet one of these three criteria: (i) participate in vigorous-intensity activity for at least 20 min per day for 3 or more days per week, (ii) participate in moderate-intensity activity or walking for at least 30 min per day for 5 or more days per week, or (iii) participate in any combination of walking and moderate- or vigorous-intensity activity for 5 or more days per week, with a minimum score of 600 MET-minutes/week. Individuals who fail to satisfy the criteria for being classified as High or Moderate in terms of PA are categorized as having a Low PA level. Based on these criteria, IPAQ features are composed of the MET score (MET), indicating PA levels corresponding to Low, Moderate, High and the IPAQ category (Low = 1, Moderate = 2, High = 3).

### 3.2. Features of Obesity

Obesity-related features are total fat mass (DW_WBT_FT) and total body fat percentage (DW_WBT_pFT) as measured with DXA. Furthermore, waist circumference (HE_wc) and prevalence of obesity (HE_obe) were evaluated using anthropometric measurements. Obesity prevalence was investigated only in subjects aged 19 years and older and was dichotomized via data preprocessing, with 0 being normal or underweight and 1 being obese. The survey also includes BMI associated with obesity, calculated as weight/height${}^{2}$ (kg/m${}^{2}$).

### 3.3. Statistical Analysis of PA and Obesity Features

In this section, we described the analysis of the PA and obesity features in respect to sarcopenia prevalence, as shown in Table 1, where statistical values of these features are compared according to gender (male and female).

#### 3.3.1. Statistical Characteristics for Gender

The statistics in Table 1 indicate that the mean age of the male and female populations is approximately the same. Males and females differ in muscle/fat mass and PA ability due to physical and physiological differences. The ASMI used to determine sarcopenia is higher in males, who had a mean value of 7.14 as compared to the 5.91 for females. However, the mean value of BMI is higher for females. Regarding the PA features, we confirmed that all values except the feature BE3_21 are higher in males. Similar to the BMI, obesity features are high in females except for HE_wc. With respect to IPAQ features, males also exhibit higher MET and engage in more intense PA than do females.

#### 3.3.2. Sarcopenic Statistics and Analysis

We used a chi-square test for categorical variables and an independent-samples *t*-test for continuous variables to determine the statistically significant differences according to sarcopenia for each gender. If the *p*-value was less than 0.05, it was judged that there was significance difference according to sarcopenia prevalence.

As evidence of age-related disease, males without sarcopenia are on average approximately three years younger than those with sarcopenia. Conversely, the BMI is smaller in sarcopenic males. Except for BE3_31, pa_mid, and pa_walk, which had a *p*-value of 0.05 or more, all PA feature values showed significant differences. These PA features tended to be higher in males without sarcopenia. Moreover, it was confirmed that males without sarcopenia have significant difference and higher feature values than do those with sarcopenia, except for DW_WBT_pFT among the obesity features and mod and low among the IPAQ features.

Similarly, females without sarcopenia are younger on average than those with sarcopenia, as in men. Furthermore, the BMI is lower in sarcopenic females. Females without sarcopenia show significant difference and higher values in BE3_11, BE3_12, BE3_21, BE3_22, pa_high, and pa_mid among the PA features. In obesity features, except for DW_WBT_pFT, nonsarcopenia females exhibit higher values. Additionally, there are significant differences among the IPAQ features such as MET, high and IPAQ.

From these statistics, we can observe that most of PA and IPAQ features show significant difference and lower values in the sarcopenic population regardless of gender. Furthermore, a similarly low value is observed in the obesity features of total fat mass and waist circumference. Sarcopenia not only causes physical discomfort and can interfere with PA but it is also related to obesity factors. As those feature values show particularly significant differences according to sarcopenia prevalence, we could establish a prediction model using ML algorithms with the aforementioned features.

## 4. Sarcopenia Prediction Modeling Using Machine Learning

Using the correlation between sarcopenia and PA, IPAQ, and obesity features discussed in the previous section, we aimed to establish a sarcopenia prediction model for elderly individuals with PA-related information. The aforementioned PA-related information is easy to obtain compared with other information measured by special devices and experts. To develop the prediction model, we used various ML techniques such as LR, SVC, KNN, ensemble learning bagging-type RF, and boosting types such as GBM, XGB, LGB, and CAT. Furthermore, we considered a deep learning model using MLP and DNNs. In addition, the importance of PA features was estimated by identifying variables that significantly affect the sarcopenic prediction model. For this, the contribution of each features was evaluated by the SHapley Additive exPlanations (SHAP) value according to each algorithm. We introduce briefly the ML algorithms used in this study with respect to algorithmic mechanisms and characteristics.

LR [37] is a popular statistical learning algorithm well-suited for solving classification problems. LR uses a logistic function to model the association between input features (independent) and a binary target variable (dependent). Interpreting the output of the logistic function as the probability of the binary variable, the LR model can predict the value of the binary variable. LR employs optimization algorithms such as gradient descent to minimize the loss function and learn model parameters.

SVC is a type of support vector machine [38] used for classification tasks. The goal is to identify the best hyperplane that can effectively separate data points belonging to different classes. This is achieved by maximizing the margin, which refers to the perpendicular distance between the hyperplane and the closest data points from each class. By maximizing the margin, SVC seeks to identify the most robust decision boundary that can generalize well to new, unseen data. SVC aims to minimize the classification error and can handle high-dimensional data effectively.

KNN [39] is a type of ML algorithm that can be used for both classification and regression tasks. It assigns a new data point to the class of its K-nearest neighbors in the training set based on the similarity between their feature vectors. KNN is a simple algorithm that can be effective for low-dimensional data but may struggle with high-dimensional data due to the “curse of dimensionality”.

Ensemble learning is an ML technique that merges multiple models to improve overall predictive accuracy. It emerged as a way to address the limitations of single models and improve the accuracy and robustness of predictions. There are several types of ensemble learning, including bagging and boosting. Bagging (bootstrap aggregating) works by creating multiple bootstrap samples (random subsets with replacement) of the original training data, training a base model on each sample and then aggregating the predictions of these models. Boosting works by iteratively training weak models on the original data, assigning higher weights to misclassified instances in each iteration and then combining the predictions of these models.

Among the bagging methods, decision tree-based RF [40] is representative. RF builds a multitude of decision trees and combines their predictions to improve accuracy and reduce overfitting. It introduces randomness into the decision tree-building process by training multiple decision trees on different bootstrap samples of the data and randomly selecting a subset of features at each split to create a diversified set of classifiers. The final output is typically the majority vote in classification tasks. RF is a popular and widely used ML algorithm owing to its high accuracy, robustness to outliers, and ability to handle high-dimensional datasets with many features.

GBM [41,42], a boosting method, combines multiple weak models to create a stronger model. It works by iteratively training models on the residuals of previous models by gradient descent optimization. A weak model is trained on the negative gradient of the loss function and added to the prediction of the previous model to update the residuals. The final prediction is obtained by adding the predictions of all weak models, with each model being trained to correct the errors of its predecessors. GBM is known for its ability to handle different data types and achieve high accuracy with relatively few iterations. There are also the XGB [43], LGB [44], and CAT [45] algorithms, which have improved accuracy, speed, and resource usage in the GBM method.

MLP [46] is a forward artificial neural network in which multiple layers of perceptrons are stacked. It is composed of an input layer, one or more hidden layers, and an output layer. Depending on the number of final output layers, classification for the desired number of classes can be achieved. The model is learned through error backpropagation using a stochastic gradient descent.

A DNN is a type of neural network architecture that contains two or more hidden layers between the input and output layers. The additional hidden layers enable the network to learn more complex representations of the input data, resulting in improved performance on a wide range of ML tasks. Hundreds of additional hidden layers have recently shown excellent performance in various applications with structures of convolutional neural networks and recurrent neural networks.

## 5. Experiments

We trained 10 sarcopenia prediction models using the aforementioned ML algorithms and evaluated them with test samples in terms of sarcopenia prediction accuracy. To derive the general performance of the model using full given data for training and validation, we conducted 4-fold cross-validation with 75% training samples and 25% test samples, which resulted in average values. We implemented the ML algorithms using the ML library and deep learning framework. PA, IPAQ, and obesity data samples as the sarcopenia features were used for ML. To evaluate the feasibility of each feature for sarcopenia prediction, learning experiments were conducted repeatedly with various feature combinations to obtain higher prediction accuracy. Considering that some obesity features, such as the total body fat mass and percentage—but not waist circumference—are difficult for individuals to measure, we compared the prediction accuracy of the models with or without obesity features in addition to PA features. In addition, each model was trained with data of both genders, male only, and female only to investigate the prediction accuracy according to gender.

We explored several hyperparameters of a total of 10 models by tuning and observed that the results from these variants were comparable. Accordingly, we applied the default parameters of the sklearn [47] or PyTorch library [48] for all models except several hyperparameters for the DNN. The DNN model was configured by four linear layers including a dropout layer with the following hyperparameters: learning rate = 0.001, batch_size = 96, and dropout_rate = 0.1. It performs binary classification by applying the sigmoid function to the last linear layer.

For data normalization, RobustScaler was used for all features; it is robust for medical data with many outliers that degrade the performance of the prediction model and also shows the highest accuracy from normalization. RobustScaler scales data using the median of the data and the interquartile range (IQR) between 25% and 75% of the data. Accordingly, it works effectively even if data are not normally distributed and helps to extract common patterns from data that contain outliers.

We plotted the learning accuracy and fitting time of each model according to the training sample ratio in order to show the process of the learning. The sample ratio, the current ratio of data learned for the total training samples, was set to 0.1, 0.325, 0.55, 0.775, and 1.

### 5.1. Prediction Performance with PA and Obesity Features

**Figure 2:**
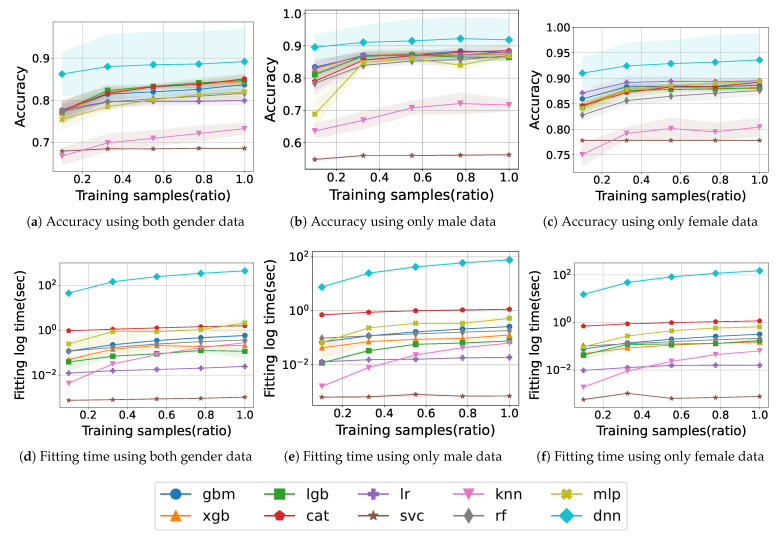
Sarcopenia prediction accuracy of ML models using PA, obesity, and IPAQ features.

First, we considered all given features, including age and BMI, in the model training. Figure 2 shows the learning curve and fitting time for model training with PA, IPAQ, and obesity features according to gender.

Figure 2a shows the accuracy according to the number of training samples and is denoted by the ratio of used samples to total training samples, when using both gender data. The traditional ML method, SVC, maintains an accuracy of approximately 0.68 without improvement, and KNN improves from 0.66 to 0.73, but both models have a lower accuracy than do the others. LR does not change significantly after the accuracy reaches 0.80. RF and MLP consistently exhibit an increasing accuracy during training and finally achieve an accuracy of 0.81 and 0.82, respectively. The boosting models also accomplish a monotonic improvement in accuracy as the training samples increase, and among them, CAT particularly exhibits the highest accuracy of 0.85 at the end. Finally, the model with the highest accuracy is DNN, which records 0.89 and demonstrates a remarkable accuracy of 0.86 from the beginning of training.

Figure 2b shows the accuracy when training with only male data of the same features. SVC does not achieve an accuracy of up to 0.6, which is lower than when not distinguishing gender. The accuracy of KNN drops from 0.72 to 0.71 at the end, and, unlike before it gradually improves. XGB and LGB show a slight dip midway into training and, finally, exhibit a maximum accuracy of about 0.87. Similarly, MLP also decreases from 0.86 to 0.83 in the middle of training, but the final accuracy reaches to 0.86. Most of the models converge to around 0.87. Meanwhile, DNN exhibits the highest accuracy, exceeding 0.91.

Figure 2c shows the accuracy when training with only female data. High accuracy is obtained by all models as compared to previously, especially SVC and KNN, which recorded low accuracy, both achieve an accuracy of over 0.77. Most models draw learning curves up to approximately 0.88. Among them, LR records the second-highest accuracy from start to finish, achieving a final score of 0.89. In particular, the DNN model shows a remarkable result, showing an increase in accuracy from 0.91 at the beginning to 0.93 at the end.

Figure 2d illustrates the fitting time trained with both gender samples. SVC is trained in a very short time, less than 10${}^{-2}$ s. The KNN model also starts with a very small fitting time and gradually increases and is trained within around 1 s like other models. In contrast, the DNN model takes a relatively long training time compared with the others, which takes 44 s up to the 0.1 training sample ratio and then increases by approximately 100 s for a total learning time of 438 s.

Figure 2e,f illustrates the fitting time trained with only male or female samples. For males, the fitting time for DNN is 8 s initially and then increases by 18 s continuously until 80 s. In the case of females, with approximately 600 more female data samples than male samples, it takes a little longer time, with a total of 150 s. The other models complete training within 1 s in either case.

We obtained the final accuracy of 0.89, 0.92, and 0.93 for both genders, male only, and female only, respectively, with obesity and PA features. The DNN model achieved the highest accuracy in all feature combinations, including obesity. We acquired higher accuracy when learning with separated male and female data rather than both data. In particular, the accuracy with only female data was higher than that with other data. The fitting time of DNN was proportional to the amount of data, whereas other models exhibited short and comparable learning times across the data amount. Furthermore, the fitting time shows a similar tendency regardless of gender datasets.

#### Accuracy Analysis for PA Features with Obesity

Table 2 shows the final prediction accuracy of different feature combinations in addition to obesity features: (i) all is a combination of all features, including PA, IPAQ, and obesity; (ii) onlyMET is a combination of PA and obesity features including only MET scores in IPAQ feature; (iii) onlyIPAQ is a combination of only IPAQ features and obesity without other PA features.

From the results of all, DNN has the highest accuracy of 0.892 for both genders, followed by the boosting models CAT, LGB, and XGB with 0.851, 0.847, and 0.844, respectively. For the male data, DNN also has the highest accuracy of 0.919, and CAT has the second-highest accuracy. For the female data, DNN exhibits an accuracy of 0.936, higher than other cases, and LR has the second-best accuracy. From the onlyMET result, DNN records the highest accuracy of 0.902, 0.916, and 0.934 for both gender, male, and female data, respectively. The boosting models and LR exhibit the second-highest accuracy. Finally, according to the results of onlyIPAQ, DNN achieves an accuracy of over 0.9 on all datasets, with 0.926 and 0.937 for males and females, respectively, showing the highest accuracy among feature combinations.

As a result, the DNN model with onlyIPAQ features shows the best performance, and the results of onlyMET and all are comparable. Given the negligible difference between the results, it can be concluded that IPAQ features, except for MET, do not have a significant impact on the training. The reason that onlyIPAQ feature combination, excluding PA features, achieves the best performance is likely due to the excess PA features interfering with learning by overfitting.

The accuracy of the models varies slightly depending on the feature combination used for training. Among them, DNN always exhibits the best accuracy. After that, most of the boosting models or LR have the second-highest accuracy. The DNN model trained with features, including PA, IPAQ, and obesity features, can achieve a high average accuracy of about approximately 90% across all feature combinations. Except for SVC and KNN for males in all and onlyMet, all remaining models improve more than this in predicting sarcopenia for separate gender classes of males or females. This increase in the accuracy of most models explains the trivial differences between male and female characteristics of feature values.

### 5.2. Prediction Performance with Only PA Features

**Figure 3:**
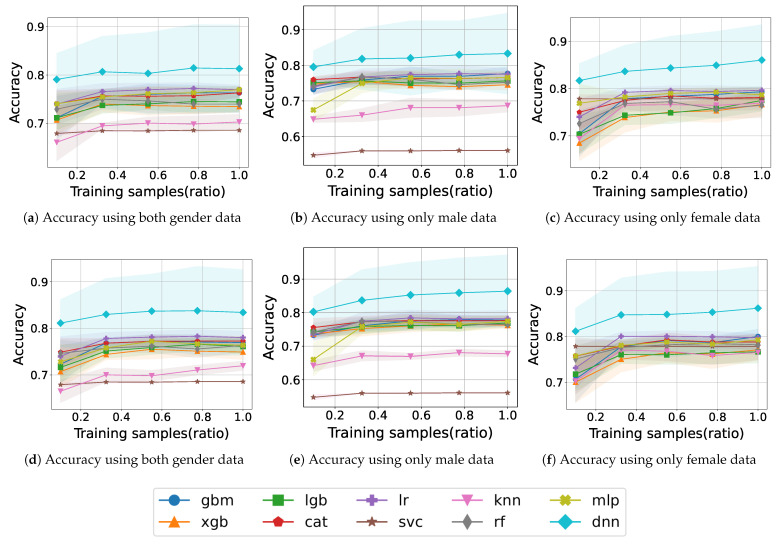
Sarcopenia prediction accuracy of ML models using PA and IPAQ features. (**a**–**c**) The model accuracy of training with PA and IPAQ features. (**d**–**f**) The model accuracy of training with PA, IPAQ, and HE_wc features.

Although it is possible to obtain high accuracy with obesity features, they are difficult to measure personally except for waist circumference. For instance, obesity features such as total body fat mass, total body fat percentage, and obesity prevalence can be measured only by special equipment or specific institutions. Therefore, it is meaningful that model training is conducted using only PA and IPAQ features in addition to basic information such as age and BMI. Figure 3 shows the learning curve for model training with PA and IPAQ features according to gender.

Figure 3a shows the accuracy when training with samples for both genders. SVC and KNN exhibit accuracy approaching 0.7, and other models converge around 0.75 without significant fluctuations. During training, we obtained an accuracy of 0.77 for LR at a 0.775 sample ratio and 0.73 for XGB at 0.55. The DNN model accomplished the best accuracy, with results exceeding 0.81.

Figure 3b shows the accuracy when training with samples for only males. The accuracy is less than 0.6 for SVC and less than 0.7 for KNN even though it increases gradually. In the boosting models, the accuracy remains almost constant during training; the XGB model has the lowest accuracy of 0.74 at the end, whereas GBM has the second-highest accuracy of 0.77. XGB and RF demonstrate a decrease in accuracy during training. The DNN shows a gradual improvement in accuracy and reaches an accuracy of approximately 0.83.

Figure 3c shows the accuracy when training with samples for only females. The accuracy of SVC starts with 0.77 but not in the lower ranks from the beginning of training. Compared with the low accuracy of some models for the male case, all models demonstrate an accuracy of 0.74 or higher from the 0.335 sample ratio. Except for CAT, RF, and MLP, the remaining models show no decreasing fluctuation and maintain or show increasing accuracy. XGB shows low accuracy from start to finish, RF ends with 0.76 which is the lowest final accuracy. Most of the models exhibit learning curves toward approximately the upper half of 0.7. The DNN model maintains an accuracy of 0.81 at the start of training and 0.86 at the end.

The absence of obesity features causes the outstanding accuracy of DNN to drop from approximately 90% to approximately 81%. In addition, other models have similar reductions even though their ranks in terms of accuracy are changed. This shows that the obesity feature is critical in sarcopenia prediction. Considering the difficulty of measuring obesity features, we conducted an additional experiment with a waist circumference feature, which is easy to measure self-measure.

Figure 3d shows the accuracy of training with samples including HE_wc for both genders. The accuracy of KNN is originally 0.7 but increases slightly to 0.72 after the addition of the waist circumference feature. Some models have fluctuations in the middle, but in the end, all models show higher accuracy than before the addition of features. There is an increase from 0.74 for LGB and 0.73 for RF to 0.76 both. The DNN model exceeds the previous final learning result of 0.81 from the start of training and reaches 0.83.

Figure 3e shows the accuracy of training with samples including HE_wc for only males. Except for SVC and KNN, the rest of the models perform better than before in most of the learning curves. The learning curves, originally within the 0.74–0.77 range, rise to the 0.76–0.77 range, i.e., the lower bound rises. In particular, the accuracy of the DNN model, which is 0.81 without waist circumference, increases to 0.86.

In Figure 3f, the learning curve when training samples including HE_wc for only females shows an increase compared to that before adding the waist circumference feature. SVC starts with high accuracy, and the final accuracy of the all models, excluding DNN, plots learning curves between 0.76 and 0.79. Similar to the learning curve before adding waist circumference, none of the models acquire particularly poor accuracy. DNN steadily increases from 0.81 to 0.86 depending on the training sample ratio.

The waist circumference feature is useful for improving accuracy in addition to PA features. Comparing the results before and after adding the waist circumference feature, we can see that most models converge to higher a accuracy than before. By the simple addition of waist circumference, the accuracy increases in both genders. Examining the learning curves, we can see that SVC and KNN have low accuracy in males, while all models in females maintain a relatively high accuracy without low accuracy. It can be confirmed through the tendency of the learning curve that gender-specific characteristics affect the learning of the model, and the fact that learning by separated gender is more accurate than when learning by both supports this. Based on this, we argue that it is preferable to build a sarcopenia prediction model separately according to gender for high prediction accuracy.

#### Accuracy Analysis for PA Features without Obesity

Table 3 shows the prediction accuracy of different feature combinations without obesity features. all, onlyMET, and onlyIPAQ were performed with the same combination of features as before except for the obesity feature. Furthermore, the performance of withHE_wc that considered the waist circumference feature in addition to the PA and IPAQ features was evaluated.

Similar to the model accuracy when obesity features were used, the accuracy increased more when only male or female gender is separately considered than when both genders are considered. For results of the all case with only PA and IPAQ features, DNN also achieved the highest accuracy, with 0.813 for both genders, 0.833 for males, and 0.860 for females. LR trained on both or female data and GBM trained on male data recorded the second-highest value, but it did not exceed 0.8. In the result of onlyMET, DNN shows an accuracy of 0.834 in males and 0.838 in females but has an accuracy of 0.812 in both. Meanwhile, GBM and LR achieve similar a accuracy as that previous. In the onlyIPAQ result, DNN also shows the highest accuracy but the lowest accuracy over other feature combinations.

The other models, except for the DNN model, failed to achieve prediction accuracy of more than 0.8 with PA features. The DNN model showed the highest accuracy in all with the highest amount of information for only PA-related information. When the waist circumference was also considered, the accuracy of the model could be improved. As a result, the respective DNN accuracy for both gender, male, and female data was 0.835, 0.864, and 0.862, respectively, which were the highest accuracy values among the PA feature combinations. By using self-measurable features, such as PA and waist circumference, we were able to achieve a significant accuracy of 84%. The increase in accuracy when separating the gender was also found in withHE_wc, with an astonishing 86% accuracy being achieved.

### 5.3. Reliability of the Prediction Model

In the case of medical data, since the frequency of occurrence of a specific disease is very low, it is difficult to obtain data of a positive class, resulting in a class imbalance phenomenon in which relatively small samples exist compared to the normal population often occurs. This feature can be also confirmed in the KNHANES data set used in this study, with sarcopenia in only 30 percent of the total. Accuracy may not accurately reflect the performance of the model when there is an imbalance between classes, so to evaluate the reliability of a more accurate model, evaluation metrics such as precision, recall and AUC are also used in the analysis. Since we aim for a model that accurately classifies patients with sarcopenia, we check the classification results for the corresponding positive label. In the same way as when measuring accuracy, for each model that has performed 4-fold cross-validation, each average value of various metrics is examined and compared taking into account for class imbalance.

#### 5.3.1. Confusion Matrix

**Figure 4:**
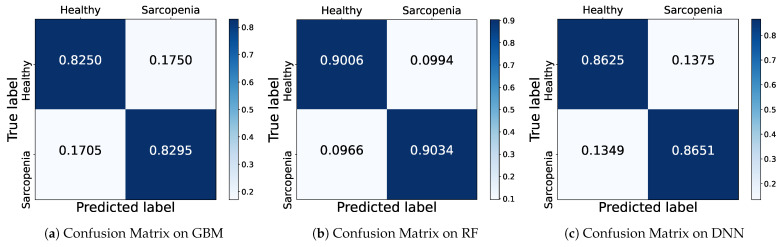
Confusion Matrix of ML models trained with withHE_wc for male.

To show how accurately the model predicted each class, we depict a confusion matrix as shown in Figure 4, a table representing the results of actual and predicted values in a 2 × 2 matrix. The rows represent the actual class (True label), and the columns represent the predicted class (Predicted label). Correct predictions against the positive and negative classes are called True Positive (TP) and True Negative (TN), respectively. When a negative is incorrectly predicted as a positive, it is called as False Positive (FP), and otherwise False Negative (FN) for an opposite case that a positive is predicted as a negative. In this case, in order to determine the prediction ratio of the model according to the number of samples of the actual class, the row values are normalized by 1.

The Figure 4 shows the confusion matrices of GBM, RF and DNN models based on withHE_wc male data which achieves the highest accuracy among all feature combinations. In the first row of the GBM confusion matrix, the model classifies a healthy sample as healthy with a probability of about 0.83 and sarcopenia with a probability of about 0.17. In the second row, the sarcopenia samples are classified as healthy with a probability of about 0.17 and sarcopenia with a probability of 0.83. The GBM predicts successfully with almost 0.83 for both samples. The RF model achieves better prediction with a probability of about 0.90 for both healthy and sarcopenia samples than the GBM. Also, the DNN classifies successfully both classes with a probability of 0.86, which is a relatively lower result than RF. Accordingly, we conjecture that the higher accuracy of the DNN is induced from imbalanced samples of sarcopenia according to the confusion matrix that confirms the exact performance for each class.

#### 5.3.2. Comparison of Other Metrics

Precision and recall can be extracted from the predicted probability for each class as in the confusion matrix. Precision is the proportion of data that the model predicted to be in the positive class that actually belonged to the positive class, and recall represents the proportion of samples that the model predicted to be positive out of samples that are actually positive. In other words, precision is a metric that focuses on how accurate the predictions of models are, and recall is a metric that focuses on how well the model detects the true positive class.

**Figure 5:**
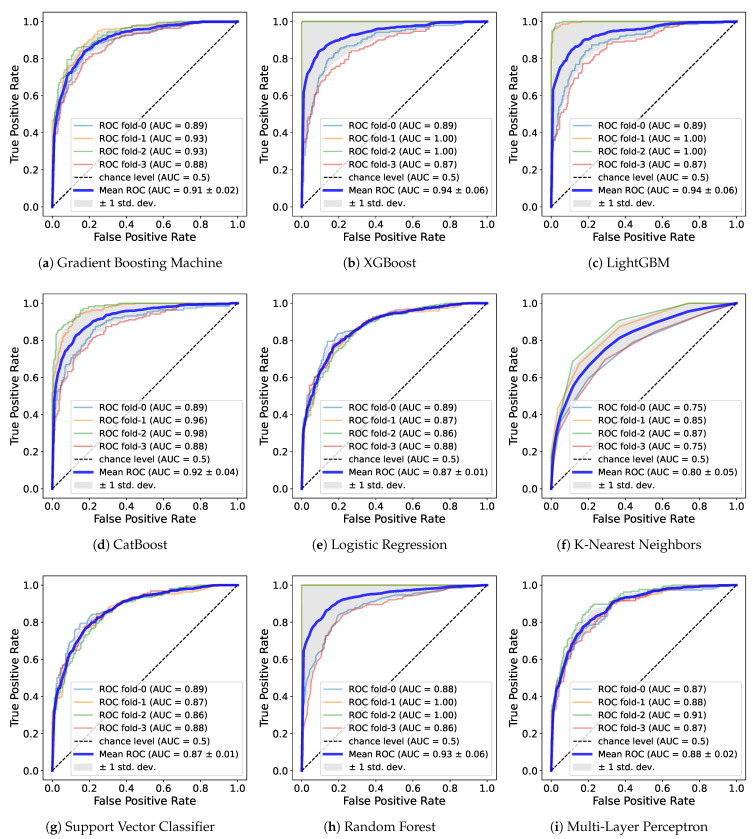

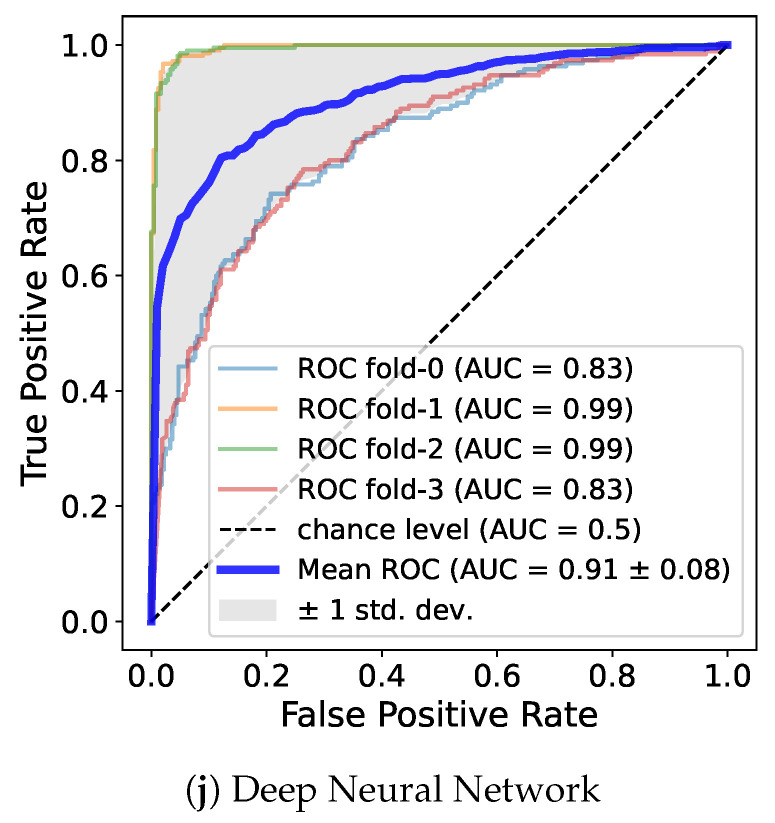
ROC curve and AUC of ML models of withHE_wc in male case.

Also, we use the ROC curve. The ROC curve is a commonly used evaluation method in binary classification and is a graph that enables judgment of whether a classification model shows good performance. Ultimately, the AUC value based on the area under the ROC curve is used as a performance metrics for classification. When a dataset exhibits class imbalance, AUC can be used to avoid models with poor predictive performance for minority classes despite high accuracy. AUC evaluates performance at all possible classification thresholds, so we can adequately evaluate model performance even on imbalanced datasets. The closer it is to 1, the better the performance; the curve plots a rectangle shape as the area below it becomes 1. Table 4 shows metrics such as precision, recall, and AUC and Figure 5 demonstrates the ROC curve for a model trained on withHE_wc for male.

While the DNN model was ranked first with the highest accuracy in the previous experiment regardless of gender, and LR or the boosting models had the second-best performance, the precision and recall showed different results. For both genders and for female, RF had the highest value, and XGB, LGB, DNN, and the rest of the boosting models all recorded values above 0.8. Similarly, in the case of males, boosting models, RF, and DNN had relatively higher values than did the other models. In particular, XGB recorded the highest value exceeding 0.9. Overall, the RF, DNN and boosting models such as XGB and LGB showed high value regardless of gender. The fact that certain models have high recall and precision in common means that samples belonging to true positive class are found well, and among the samples predicted by the model to be positive, the proportion of actually positive samples predicted is high. In other words, it can be said that the model effectively predicts the correct class.

The AUC to evaluate the overall performance of the model regardless of data imbalance is as follows. In both genders, RF had the highest, exceeding 0.91, followed by XGB, LGB, CAT, DNN, and GBM. In males, LGB, XGB, and RF recorded approximately 0.93, the highest among the genders. CAT, DNN, and GBM also exceeded 0.9. In females, LGB, RF, and XGB was close to 0.9, and the boosting models and DNN showed values in the upper half of 0.8. It was confirmed that RF performs well in both data sets and LGB performs well in each gender. Similar to the accuracy of training with withHE_wc, the AUC was highest in male. Through AUC, a reliable measure of performance, it can be seen that PA features including waist circumference more effectively predicted sarcopenia. Additionally, regardless of gender, most models showed high values close to 0.9, so those features are useful for learning.

Similar to the results in precision and recall, boosting models, RF, and DNN were ranked at the top in AUC. All of these models, regardless of gender, had an AUC value of at least about 0.85. These models performed similarly well when measured across metrics, indicating that they predicted sarcopenia more reliably than did the other models. Depending on gender, some models recorded high AUC values over 0.9, and the lowest value was also above 0.85, which significantly exceeds the 0.8 of existing prediction models based on intake and blood cell count information [24]. Consequently, we can argue that our predictive models based on only PA features and waist circumference are competitive. Furthermore, it was confirmed that the RF, DNN, and boosting models such as XGB and LGB show strong performance especially for these feature combinations. This result, despite the use of the default hyperparameters, is expected to increase the AUC value of the ML model through hyperparameter tuning. In particular, a DNN model that shows a significant result even with the simple structure of several linear layers has more tuning factors than do other models, so it is highly likely to be improved.

## 6. Discussion

### 6.1. Key Feature Selection Based on SHAP Value

**Figure 6:**
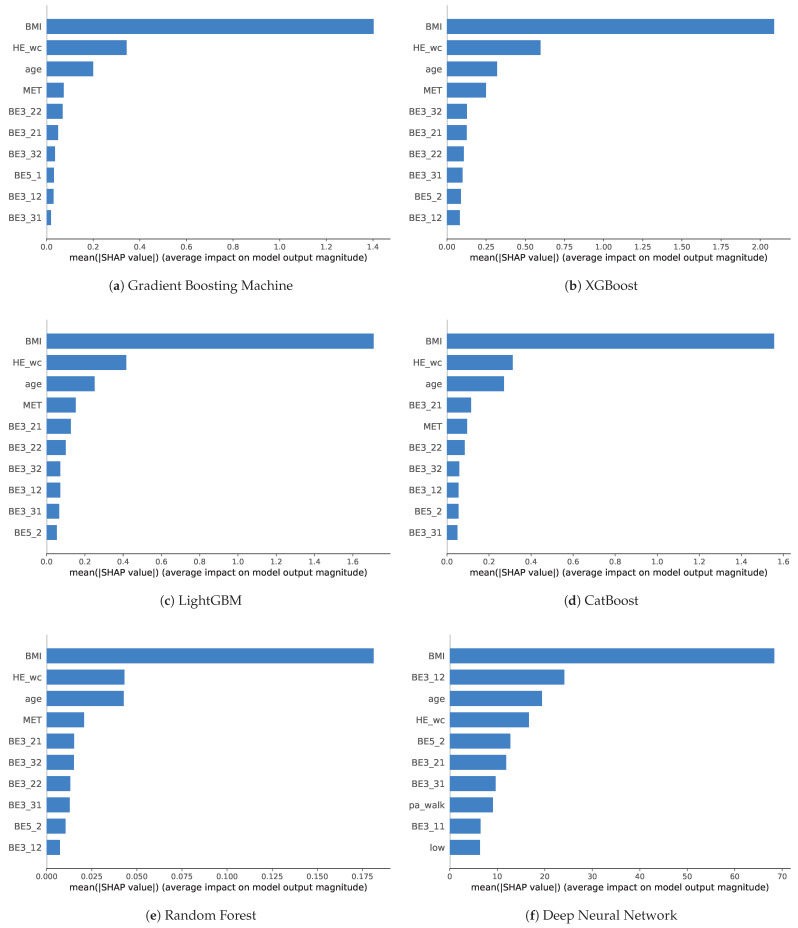
SHAP plots which show the feature importance of the top 10 features trained with withHE_wc for both in each ML model.

We analyzed the impact of features on the ML algorithms. To examine the importance of features, we analyze feature importance with SHAP for models with high performance. We did not separate male and female to determine the general feature importance. Through SHAP values, it is possible to know how much each feature influences the results of models and express it as a plot that shows the importance of each feature. After identifying the importance of features, we ordered the feature importance according to the model and scored the frequency of use to comprehensively identify features that have a significant influence on sarcopenia prediction. It can be inferred that features with high importance in common across models are effective features for predicting sarcopenia. This extracts features that are relatively effective in predicting sarcopenia among PA features. Figure 6 shows the feature importance of training using withHE_wc for both genders. We selected 6 among 10 models with AUC threshold values of 0.85, which exhibited a robust performance on the $withHE_{w}c$ data regardless of gender such as GBM, XGB, LGB, CAT, RF, and DNN. Furthermore, these 6 models showed higher AUC values, above 0.9, as compared with the others, even for certain genders. As a consequence, we measured SHAP values only for these 6 models and extracted features that effectively influenced the models.

For the boosting models, including GBM, XGB, LGB, and CAT, the BMI showed the most significant importance, followed by waist circumference and age. Although the importance was smaller than before, MET was the fourth most important in all models except for CAT, which had the number of days of moderate PA. After this, the order and importance of features differed according to the model. Various durations, number of days of PA, and number of days of flexibility exercise were generally ranked in the top 10. Overall, similar features were ranked high for each boosting models, and their importance also showed a similar tendency. This meant that the features act with similar importance in the boosting model. In RF, SHAP value was overwhelmingly high in BMI, followed by HE_wc and age, which were almost equal in importance. It cab be also seen that MET and other PA features have relatively low importance. However, feature importance in the DNN model was slightly different from that in the ensemble models. While BMI, waist circumference, and age ranked similarly high in terms of importance, the duration of vigorous PA was ranked second. Most of the features that were related to PA were located in the upper ranks unlike the other models, even if MET did not even rank in the top 10. The number of days of flexibility exercise ranked fifth, the number of days of moderate PA ranked sixth, and the number of days of walking ranked seventh and thus had a certain level of high importance. Features related to numbers of days of PA were mostly seen rather than duration. Meanwhile, the corresponding importance was comparable in contrast to that of other models. Furthermore, the walking rate and Low not seen in other models were found.

Comparing the absolute values of the mean shape values is difficult because each value is relative across models. Instead, by scoring the top 10 most important features used in the model that perform well on performance measures, it is possible to briefly identify important features that are common. Table 5 shows the feature score with a maximum value 6, which is the sum of used features marked with 1 and unused features marked with 0 for each model.

Age, BMI, duration of vigorous PA, number of days of moderate PA, number of days of walking, and waist circumference were used in all models. Because age, BMI, and waist circumference showed particularly high importance in the ensemble models, these features can be interpreted as having a high causal relationship with sarcopenia. No IPAQ features received a score of 6, but MET scores contributed highly to most of the models. The duration of days of moderate PA, duration of walking, and number of days of flexibility exercise received a total of 5 points as the next most important factors. After that, there was no feature with a score of from 2 to 4. The number of days of strength exercise utilized for GBM obtained a score of 1. There were also number of days of vigorous PA, walking rate, and Low, which were selected only by DNN. Vigorous PA rate and moderate PA rate and the rest of the IPAQ features were not in the top 10 for all models.

Note that PA information and body information such as age, BMI, and waist circumference helps predict sarcopenia. Among PA information, features related to moderate PA or walking are the most important, and flexibility exercise are also related. MET scores, a numerical measure of PA, also play an important role. Although the feature importance differs for each model, we can extract key features for sarcopenia prediction through scoring, which can be used for further analysis and future research.

### 6.2. Limitations and Future Work

Some limitations of our study should be considered. First, we used slightly outdated data, the KNHANES data from 2008 to 2011, because the investigation for judging sarcopenia was not conducted in other years. Accordingly, our results are limited to the specific period, but the outcomes are probably not much different for other years. However, we will continue further study on the data gathered from private healthcare centers which includes BIA data for sarcopenia diagnosis [30,32].

Second, we only investigated the general performance of the sarcopenia prediction based on default parameter values of libraries in this study. ML models can achieve better performance after adjustment of hyperparameter values to fit the model against the data. Therefore, there is still the possibility to improve accuracy slightly with optimized parameters in each model.

We have identified ML models that show feasible performance only with the PA data that can be measured easily even by the elderly. To further improve the performance of the model, we now plan to add more data features of the living environment related to the PA together with hyperparameter tuning. Additionally, we will use real-time sensory data from wearable devices and smartphones for multimodal learning of the prediction models.

## 7. Conclusions

Previously studies have investigated the relation between sarcopenia and PA. However, efforts to make a prediction model using the PA has not been conducted, since even though it is critical to obtain nation-wide samples related to sarcopenia from elderly people, sarcopenia was not recognized as a disease in Korea until last year.

Therefore, we developed a sarcopenia prediction model based on PA that is more practical than are other models based on medical measurement. We used the the KNHANES data containing the sarcopenia information that has not been explored in relation to physical activity. We implemented prediction models with various machine learning algorithms and compared them in terms of performance. Additionally we analyzed the feature importance of the models and proposed key PA features for the prediction model.

From experimental results, we observed that the obesity features related to the PA were effective for the prediction model; for instance, the DNN accuracy on average was close to 90% across all data combinations and dropped to 81% without obesity. Simple waist circumference information was helpful in improving the accuracy to 84%. This model accuracy could be improved by separating the training sets according to gender.

For further analysis on the performance, we investigated several metrics such as precision, recall, and AUC. The RF and boosting models showed better performance for precision and recall metrics, as compared with DNN, with the highest accuracy due to the imbalanced samples of sarcopenia. For this, AUC values for reliability showed that RF outperformed others in mixed data, while the LGB did better in separate data sets. Considering overall metrics, we can conclude that GBM, XGB, LGB, CAT, RF, and DNN are effective algorithms for modeling sarcopenia prediction based on the PA.

For the key PA features, we analyzed feature importance of models using SHAP values and created a score table to derive key PA features from the best models, GBM, XGB, LGB, CAT, RF, and DNN. Accordingly, we found the key features with a score above 5, such as the duration of vigorous PA, number of days of moderate PA and walking, flexibility exercise, and MET score.

From this study, we can conclude that significant predictive performance can be achieved through ML only with PA features and without the other medical features preferred in previous studies. This result represents important evidence that has encouraged us to develop an AI healthcare system integrated with smart PA sensors for sarcopenia management in future work.

## Figures and Tables

**Table 1:** Statistics for sarcopenia features.

	Male (n = 1725)				Female (n = 2333)			
Characteristic	Total	Nonsarcopenia (n = 966)	Sarcopenia (n = 759)	*p* Value	Total	Nonsarcopenia (n = 1816)	Sarcopenia (n = 517)	*p* Value
Age	71.74 ± 4.58	70.74 ± 4.32	73.0 ± 4.6	<0.001	72.09 ± 4.73	71.77 ± 4.65	73.2 ± 4.84	<0.001
ASMI	7.14 ± 0.82	7.70 ± 0.55	6.41 ± 0.44	<0.001	5.91 ± 0.67	6.15 ± 0.53	5.06 ± 0.27	<0.001
BMI	23.08 ± 2.91	24.57 ± 2.42	21.17 ± 2.33	<0.001	24.08 ± 3.43	24.79 ± 3.3	21.57 ± 2.61	<0.001
PA features								
Number of days of Vigorous PA (BE3_11)	0.74 ± 1.81	0.83 ± 1.86	0.61 ± 1.72	0.010	0.54 ± 1.57	0.58 ± 1.62	0.41 ± 1.4	0.017
Duration of Vigorous PA (BE3_12)	26.78 ± 70.98	30.58 ± 72.63	21.94 ± 68.56	0.011	23.68 ± 79.09	25.74 ± 83.11	16.47 ± 62.49	0.006
Number of days of Moderate PA (BE3_21)	1.38 ± 2.37	1.50 ± 2.43	1.22 ± 2.28	0.014	1.41 ± 2.4	1.53 ± 2.47	0.99 ± 2.09	<0.001
Duration of Moderate PA (BE3_22)	44.67 ± 96.15	49.15 ± 100.59	38.97 ± 89.92	0.027	37.72 ± 89.4	41.61 ± 94.53	24.07 ± 66.7	<0.001
Number of days of Walking (BE3_31)	4.72 ± 2.72	4.77 ± 2.68	4.67 ± 2.77	0.480	3.96 ± 2.93	3.97 ± 2.92	3.95 ± 2.96	0.891
Duration of Walking (BE3_32)	74.29 ± 87.27	79.49 ± 90.42	67.67 ± 82.67	0.005	45.36 ± 58.38	44.73 ± 55.91	47.6 ± 66.32	0.323
Number of days of flexibility exercise (BE5_2)	1.59 ± 2.12	1.77 ± 2.16	1.35 ± 2.04	<0.001	1.12 ± 1.87	1.13 ± 1.88	1.06 ± 1.81	0.421
Number of days of strength exercise (BE5_1)	0.87 ± 1.7	1.02 ± 1.8	0.68 ±1.54	<0.001	0.24 ± 0.95	0.24 ± 0.96	0.23 ± 0.94	0.772
Vigorous PA rate (pa_high)	208 (16%)	133 (14%)	75 (10%)	0.014	211 (9%)	177 (10%)	34 (7%)	0.027
Moderate PA rate (pa_mid)	219 (13%)	131 (14%)	88 (12%)	0.223	288 (12%)	244 (13%)	44 (9%)	0.003
Walking rate (pa_walk)	921 (53%)	525 (54%)	396 (52%)	0.369	890 (38%)	687 (38%)	203 (39%)	0.554
Obesity features								
Total fat mass (DW_WBT_FT)	14,352 ± 4894.32	15,412.31 ± 4800.08	13,004.04 ± 4679.41	<0.001	18,830.05 ± 5687.49	19,370.35 ± 5750.55	16,932.21 ± 5023.65	<0.001
Total fat percentage (DW_WBT_pFT)	22.58 ± 5.44	22.72 ± 5.11	22.41 ± 5.85	0.250	33.98 ± 5.97	33.89 ± 5.88	34.31± 6.28	0.169
Waist circumference (HE_wc)	84.55 ± 9.06	88.05 ± 7.73	80.10 ± 8.66	<0.001	83.13 ± 9.72	84.73 ± 9.42	77.48 ± 8.6	<0.001
Prevalence of obesity (HE_obe)	417 (24%)	381 (39%)	36 (5%)	<0.001	876 (38%)	825 (45%)	51 (10%)	<0.001
IPAQ features								
MET scores (MET)	2987.1 ± 4189.29	3239.69 ± 4307.76	2665.62 ± 4013.36	0.004	2193.85 ± 4131.37	2316.56 ± 4308.81	1762.8 ± 3405.05	0.002
High (high)	488 (28.3%)	294 (30.4%)	194 (25.6%)	0.026	430 (18.4%)	361 (19.9%)	69 (13.3%)	<0.001
Moderate (mod)	632 (36.6%)	354 (36.6%)	278 (36.6%)	0.994	795 (34.1%)	607 (33.4%)	188 (36.4%)	0.214
Low (low)	605 (35.1%)	318 (32.9%)	287 (37.8%)	0.034	1108 (47.5%)	848 (46.7%)	260 (50.3%)	0.149
IPAQ category (IPAQ)	1.932 ± 0.793	1.975 ± 0.796	1.878 ± 0.787	0.039	1.709 ± 0.758	1.732 ± 0.771	1.631 ± 0.708	0.003

The features are categorized into three types, PA, obesity, and IPAQ features by colored rows in addition to general information such as age, ASMI and BMI. The results are presented as mean ± standard or as percentages with corresponding numbers. All PA features are the results measured for one week. The IPAQ category is the result of classification as *low* = 1, *moderate* = 2, and *high* = 3.

**Table 2:** Model accuracy with PA, obesity, and IPAQ features.

	PA, Obesity, and IPAQ Features								
	All			onlyMET			onlyIPAQ		
Algorithms	Both	Male	Female	Both	Male	Female	Both	Male	Female
GBM	0.837	0.879	0.886	0.836	0.884	0.888	0.836	0.886	0.888
XGB	0.844	0.875	0.880	0.847	0.871	0.883	0.844	0.872	0.881
LGB	0.847	0.864	0.882	0.847	0.868	0.884	0.849	0.875	0.888
CAT	0.851	0.885	0.892	0.851	0.881	0.892	0.851	0.885	0.895
LR	0.799	0.881	0.895	0.801	0.878	0.898	0.803	0.885	0.901
KNN	0.732	0.717	0.805	0.728	0.721	0.801	0.778	0.816	0.851
SVC	0.686	0.562	0.778	0.686	0.562	0.778	0.804	0.872	0.884
RF	0.817	0.869	0.876	0.830	0.873	0.876	0.840	0.877	0.885
MLP	0.820	0.869	0.895	0.818	0.876	0.891	0.850	0.882	0.902
DNN	**0.892**	**0.919**	**0.936**	**0.902**	**0.916**	**0.934**	**0.909**	**0.926**	**0.937**

Numbers in bold indicate the highest values of prediction accuracy.

**Table 3:** Model accuracy with PA and IPAQ features.

	PA + IPAQ Features											
	All			onlyMET			onlyIPAQ			withHE_wc		
Algorithms	Both	Male	Female	Both	Male	Female	Both	Male	Female	Both	Male	Female
GBM	0.763	0.777	0.793	0.760	0.775	0.790	0.755	0.779	0.797	0.769	0.778	0.799
XGB	0.735	0.746	0.766	0.736	0.750	0.763	0.734	0.742	0.774	0.749	0.762	0.771
LGB	0.744	0.757	0.775	0.750	0.746	0.775	0.740	0.759	0.779	0.761	0.769	0.766
CAT	0.763	0.766	0.781	0.763	0.769	0.783	0.756	0.765	0.785	0.772	0.776	0.788
LR	0.770	0.775	0.796	0.769	0.774	0.794	0.762	0.776	0.795	0.780	0.782	0.797
KNN	0.703	0.687	0.769	0.704	0.691	0.769	0.729	0.736	0.767	0.720	0.677	0.765
SVC	0.686	0.561	0.778	0.686	0.561	0.778	0.758	0.775	0.787	0.686	0.561	0.778
RF	0.740	0.752	0.765	0.740	0.748	0.769	0.734	0.748	0.758	0.763	0.765	0.782
MLP	0.770	0.766	0.787	0.765	0.765	0.791	0.762	0.779	0.794	0.763	0.774	0.793
DNN	**0.813**	**0.833**	**0.860**	**0.812**	**0.834**	**0.838**	**0.797**	**0.805**	**0.825**	**0.835**	**0.864**	**0.862**

Numbers in bold indicate the highest values of prediction accuracy.

**Table 4:** Performance metrics in case of withHE_wc for male.

	withHe_wc											
	Both				Male				Female			
Algorithms	Accuracy	Precision	Recall	AUC	Accuracy	Precision	Recall	AUC	Accuracy	Precision	Recall	AUC
GBM	0.769	0.796	0.795	0.860	0.778	0.829	0.825	0.907	0.799	0.824	0.829	0.848
XGB	0.749	0.865	0.862	0.910	0.762	**0.905**	**0.902**	0.935	0.771	0.897	0.897	0.894
LGB	0.761	0.835	0.832	0.897	0.769	0.891	0.888	**0.936**	0.766	0.878	0.880	**0.897**
CAT	0.772	0.822	0.819	0.888	0.776	0.852	0.849	0.924	0.788	0.844	0.847	0.877
LR	0.780	0.781	0.782	0.839	0.782	0.798	0.795	0.873	0.797	0.780	0.795	0.796
KNN	0.720	0.754	0.758	0.791	0.677	0.748	0.744	0.805	0.765	0.785	0.800	0.785
SVC	0.686	0.782	0.782	0.839	0.561	0.792	0.788	0.872	0.778	0.634	0.777	0.787
RF	0.763	**0.892**	**0.890**	**0.912**	0.765	0.903	0.901	0.934	0.782	**0.903**	**0.907**	0.896
MLP	0.763	0.783	0.783	0.844	0.774	0.804	0.801	0.882	0.793	0.794	0.803	0.814
DNN	**0.834**	0.836	0.832	0.869	**0.864**	0.865	0.862	0.910	**0.862**	0.864	0.863	0.858

Numbers in bold indicate the highest values of prediction accuracy, precision, recall, and AUC.

**Table 5:** Score of key features across the sarcopenia prediction models.

	withHE_wc						
Characteristic	GBM	XGB	LGB	CAT	RF	DNN	Score
Age	1	1	1	1	1	1	6
BMI	1	1	1	1	1	1	6
PA features							
Number of days of Vigorous PA	0	0	0	0	0	1	1
Duration of Vigorous PA(min)	1	1	1	1	1	1	6
Number of days of Moderate PA	1	1	1	1	1	1	6
Duration of Moderate PA(min)	1	1	1	1	1	0	5
Number of days of Walking	1	1	1	1	1	1	6
Duration of Walking(min)	1	1	1	1	1	0	5
Number of days of flexibility exercise	0	1	1	1	1	1	5
Number of days of strength exercise	1	0	0	0	0	0	1
Vigorous PA rate	0	0	0	0	0	0	0
Moderate PA rate	0	0	0	0	0	0	0
Walking rate	0	0	0	0	0	1	1
Obesity features							
Waist circumference	1	1	1	1	1	1	6
IPAQ features							
MET scores	1	1	1	1	1	0	5
High	0	0	0	0	0	0	0
Moderate	0	0	0	0	0	0	0
Low	0	0	0	0	0	1	1
IPAQ category	0	0	0	0	0	0	0

The features are categorized into three types, PA, obesity, and IPAQ features by colored rows in addition to general information such as age and BMI.

## Data Availability

Data by Korea National Health and Nutrition Examination Survey (KNHANES) are publicly available at https://knhanes.kdca.go.kr/knhanes/eng/index.do (accessed on 21 April 2023).
